# Genital myiasis associated with genital piercing. Case report

**DOI:** 10.1590/1516-3180.2017.0138290517

**Published:** 2017-11-09

**Authors:** Daniel Melecchi Freitas, Flavio Aranovich, José Nicolau Olijnyk, Renan Lemos

**Affiliations:** I Professor, Department of Urology, Hospital Conceição, Porto Alegre (RS), Brazil.; II Resident, Department of Urology, Hospital Conceição, Porto Alegre (RS), Brazil.

**Keywords:** Myiasis, Penile diseases, Body piercing

## Abstract

**CONTEXT:**

Myiasis is caused by larval infestation that usually occurs in exposed wounds. *Dermatobia hominis* is the most common fly species responsible for this parasitic infection. Genital piercing is an ornamental practice used in certain social circles. At placement, it transverses the skin surface and, as such, may be related to complications.

**CASE REPORT:**

We report a case of a 31-year-old man with a history of wound infection secondary to genital piercing who was exposed to an environment with flies, leading to myiasis. Mechanical removal and systemic antiparasitic drugs are possible treatments for myiasis. However, prevention that includes wound cleaning and dressing is the best way to avoid this disease.

**CONCLUSIONS:**

Genital piercing can lead to potential complications and myiasis may occur when skin lesions are not properly treated.

## INTRODUCTION

Although adornment with genital piercings has been increasing over recent years, the clinical implication of this practice is still under debate.^1^ Minor and major problems, such as secondary infections, have been reported on an increasing scale.^2^ Moreover, subjects who have undergone this procedure and develop any complication seek medical advice late.^3^

Myiasis is a parasitic infestation in body tissues caused by flies’ larvae. It occurs in healthy tissues (primary myiasis) or wounded tissues (secondary myiasis). *Dermatobia hominis* is the most common species of fly that parasitizes humans. The larvae are more frequently found in infected areas that present necrotic tissue.^4^

The treatment for myiasis may range from local extraction of larvae to systemic or topical use of antiparasitic drugs, depending on the severity of the infestation. We sought to report a case of secondary myiasis in a patient who developed wound infection after genital piercing. To the best of our knowledge, this is the first case reporting this medical condition (Table 1).

**Table 1:** Search of the literature in medical databases for case reports on genital myiasis associated with genital piercing. The search was conducted on February 12, 2017

Database	Search strategies	Papers found	Related papers
MEDLINE (via PubMed)	“genital myiasis” AND “genital piercing” AND case reports (publication type)	0	0
MEDLINE (via PubMed)	“genital myiasis” OR “genital piercing” AND case reports (publication type)	36	0

## CASE REPORT

A 31-year-old man came to the emergency room (ER) complaining about genital ulcers and diffuse erythema and edema in the pubic and genital area. The patient had a history of piercing placement at the base of the penis one month previously. He reported that a wound infection at the piercing site developed seven days after the procedure. At that time, the patient extracted the piercing at home and did not take any medication to treat the infection. He started then to go to sleep without clothes because of pain at the site of infection. He also reported that he kept the windows of his bedroom opened to be able to tolerate the bad smell. Two weeks later, the patient noticed an erythematous area at the pubis and genitalia that itched. He also saw some black spots and small ulcers at the previous piercing site. He described episodes of acute stabbing pain, in these areas.

**Figure 1:**
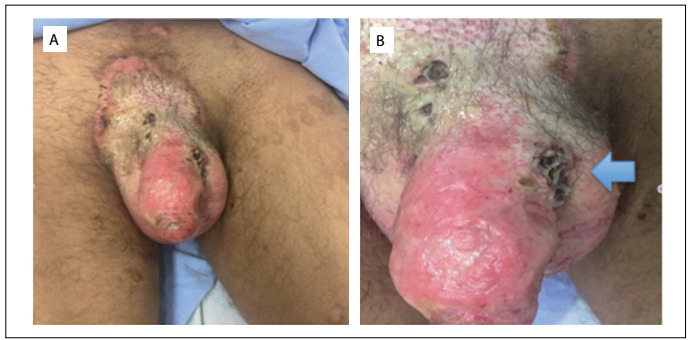
Aspect of the genitals at presentation to the emergency room. A. Ulcers. B. Arrow shows site where genital piercing had previously been placed.

**Figure 2:**
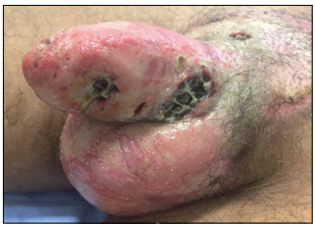
Genital ulcers following infection for genital piercing. Several larvae found inside genital ulcers.

During physical examination, his vital signs (blood pressure, pulse, respiratory rate and temperature) were within normal limits. Examination of his genitals showed that there was a large area of erythematous plaque in the pubic region with desquamation and pustules. Four necrotic ulcers were found; the largest was at the base of the penis with a diameter of 4 cm, where the urogenital piercing was previously inserted (Figures 1A and B). Inside the ulcers, several larvae were found (Figure 2). Laboratory tests demonstrated that blood parameters were normal and sexual transmitted disease tests were all negative.

**Figure 3:**
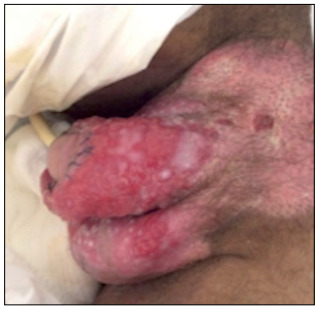
Postoperative appearance after debridment and extraction of myiasis in genitals.

The patient was taken to the operating room for surgical debridement and extraction of the myiasis under regional anesthesia. Given the large number of larvae, systemic ivermectin was prescribed after the procedure, along with antibiotics and local dressings. The patient was discharged on day seven with use of topical antibiotics and corticoids to treat and avoid new infection and decrease inflammatory reaction (Figure 3).

## DISCUSSION

Genital myiasis is a rare medical condition caused by infestation with fly larvae. It is related to poor hygiene conditions and is more prevalent in rural zones. Although uncommon, its association with genital wounds, such as those caused by genital piercing, needs to be taken into account nowadays.

In addition to genital myiasis, genital piercing can also lead to other types of complications such as bleeding, skin irritation, urinary and sexual problems.^3^^,^^5^^,^^6^ However, the lack of reporting probably stems from the fact that people with genital piercings usually seek non-medical care after complications occur.

Some studies have demonstrated that genital myiasis can mimic inflammatory or ulcerous lesions.^4^^,^^7^^,^^8^ In fact, this parasitic infestation has been associated with certain urogenital diseases. For example, Tavares et al. reported a case of a man who underwent emergency penectomy due to extensive larval infestation.^7^ In that case, myiasis was also associated with penile squamous cell carcinoma. In another case, an infectious furunculoid scrotal lesion that was thought to be neoplastic was actually caused by myiasis.^8^ In our case, the patient underwent genital piercing with subsequent wound infection. The patient presented with a wound infested by larvae inside penile ulcers.

Although cases of penile myiasis had previously been published, the correlation with genital skin lesions caused by piercing had never been reported before. Genital piercing is a form of cultural expression that can lead to several complications. Furthermore, some cases of sexually transmitted disease, like human immuno-deficiency virus infection and hepatitis, have been associated with this practice.^9^ Thus, given that genital piercing causes a skin lesion at the placement site, certain hygiene conditions need to be achieved in order to avoid wound infection and exposure to flies.

There are several ways to treat myiasis. Regarding genital myiasis, mechanical removal of larvae is the most frequent method.^10^ In general, wound closure that makes it impossible for the larvae to breath, thus causing larval death, is a common treatment. Recently, use of systemic or topical antiparasitic drugs has been described, for example ivermectin. This is a broad-spectrum antiparasitic medication that is used in selected cases that present unusual and widespread infestation and in immunocompromised patients.^11^ Nevertheless, wound closure and mechanical removal are much more cost-effective in uncomplicated cases.

The purpose of this case report was to draw attention to possible medical issues relating to genital piercing, which is a form of cultural expression. Firstly, genital piercing can lead to potential complications. A recent case series reported that almost 20% of subjects who underwent piercing in the genitalia experienced complications.^12^ Secondly, penile myiasis should be suspected in patients with genital ulcers who have low socioeconomic status and poor hygiene habits. Thirdly, although antiparasitic drugs such as ivermectin are well tolerated and have a broad spectrum, their use should only be considered in selected cases. Fourthly, prevention of infestation through cleaning and dressing of wounds is fundamental.

## CONCLUSIONS

In conclusion, among subjects who undergo genital piercing, myiasis infestation may potentially be associated with wound infection secondary to the procedure. Doctors need to be aware of this complication so as to be able to provide the best treatment for this condition and give medical counseling about the potential problems of genital piercing.
